# Network-dependent cortical thickness reductions following chronic methamphetamine use

**DOI:** 10.1017/S0033291725102067

**Published:** 2025-10-03

**Authors:** Yunkai Sun, Jun Wang, Jinsong Tang, Yanhui Liao

**Affiliations:** Department of Psychiatry, Sir Run Run Shaw Hospital, Zhejiang University School of Medicine, Hangzhou, Zhejiang, P. R. China

**Keywords:** addiction, brain network, cortical thickness, methamphetamine, MRI

## Abstract

**Background:**

Cortical thickness reductions associated with chronic methamphetamine use exhibit a non-uniform spatial distribution across brain regions. A potential neurobiological mechanism underlying for this heterogeneous pattern may involve the structural and functional organization of cortical connectivity networks, which could mediate the propagation of neuroanatomical alterations. Here, we aimed to explore how brain network architecture constrains cortical thickness alterations and their clinical relevance.

**Methods:**

The 3D-T1 images were acquired from 139 patients with methamphetamine use disorder (MUD) and 119 sex- and age-matched healthy controls. We first characterized distributed cortical thinning patterns in patients with MUD, then evaluated the relationships between regional atrophy and (1) multimodal nodal centrality measures (structural, morphological, and functional) and (2) atrophy profiles of structural connected neighbors. Individual network-weighted cortical abnormality maps were used to identify distinct MUD biotypes and related to clinical features through k-means clustering and partial least squares regression.

**Results:**

Cortical thinning patterns demonstrated significant associations with nodal centrality across all modalities, as well as cortical thinning of connected neighbors revealing a network-dependent atrophy architecture. Fronto-temporal regions emerged as critical epicenters, showing both high nodal centrality and strong correlations with connected neighbors’ thinning severity. We found that the individual differences in network-weighted cortical abnormality corresponded to clinical symptom variability, and distinguished two MUD biotypes associated with drug use.

**Conclusions:**

Our findings suggest that cortical thinning in MUD is influenced by the brain connectome architecture, providing a mechanistic framework for understanding individual variability in addiction progression.

## Introduction

Methamphetamine use disorder (MUD) has emerged as a critical public health crisis and a leading contributor to drug-related mortality (Xing et al., 2024). Chronic methamphetamine exposure induces severe mental and physical health consequences, marked by widespread cortical thinning in critical brain regions, including the prefrontal, cingulate, parietal, and temporal cortices (Koester et al., 2012; Nie et al., 2020; Petzold et al., 2022). Neuroimaging studies demonstrate that the severity of cortical volume loss escalates with both duration and cumulative drug exposure, establishing this structural decline as a progressive neuropathological process (Joo et al., 2023; Lyoo et al., 2015; Nie et al., 2021; Ruan et al., 2018). However, the mechanisms driving the regionally selective vulnerability of cortical architecture in MUD remain unresolved. Identifying the neurobiological determinants of this patterned morphological degeneration could reveal critical therapeutic entry points for halting disease progression.

Emerging evidence suggests that the formation and maintenance of addiction are driven by disruptions in brain connectome networks (Joutsa et al., 2022; Zhang & Volkow, 2019). Patterns of cortical thinning in MUD are highly organized and circumscribed by specific networks, supporting the notion that connectome architecture plays a significant role in the pathological process. For instance, brain volume loss in MUD appears to be highly organized and confined to specific functional networks such as default mode network (DMN) (Joo et al., 2023). Another study reported that cortical thinning and the breakdown of white matter integrity occur concurrently in patients with MUD (Lyoo et al., 2015). The functional connections of region showing structure alterations and the medial prefrontal cortex could predict relapse status (Geng et al., 2017). These findings support that brain network organization may be linked to structural alterations in MUD.

Network-dependent hypothesis supported that the morphological abnormalities observed in the brain may lie in the influence of the brain network or connectome (Jucker & Walker, 2018; Seguin, Sporns, & Zalesky, 2023). Specifically, neural connections might help spread pathological processes. This occurs by transporting toxic markers to distant cortical areas. Alternatively, if the network architecture is impaired, it can disrupt normal communication between brain regions (Wannan et al., 2019). Under this hypothesis, various models have been developed to explore the impact of brain networks on regional morphological changes under diseased conditions (Zhou, Gennatas, Kramer, Miller, & Seeley, 2012). One such model is the nodal stress model, which hypothesizes that highly interconnected regions appear to be more vulnerable to structural damage due to activity-related ‘wear and tear’ metabolic demands (Brown et al., 2019). For example, studies show that progressive cortical atrophy is spatially correlated with node centrality in several diseases. These include neurodegenerative diseases such as Parkinson’s disease (Zeigha et al., 2015) and Alzheimer’s disease (Zhou et al., 2012). Psychiatric conditions such as major depressive disorder (Ha et al., 2023) and schizophrenia (Georgiadis et al., 2024) also show this correlation. In addition, studies believed that white matter connectome architecture can also contribute to the progression of atrophy, where connected regions exhibit similar atrophy patterns (here, termed nodal-neighbor common atrophy model). This model has revealed regional atrophy was linked to atrophy of connected neighbors under conditions such as schizophrenia (Shafiei et al., 2020) and frontotemporal dementia (Shafiei et al., 2023). Network-dependent hypotheses provide a valuable framework for exploring the development of cortical morphology in healthy individuals, the progression of diseases, and their heterogeneity (Jiang et al., 2024; Li et al., 2024; Liang et al., 2024). However, limited research has focused on the role of these models in cortical morphology alterations associated with MUD.

In this study, we aimed to explore the associations between cortical thickness reduction in individuals with MUD and the healthy brain connectome, delineating a network-dependent spatial pattern of cortical thickness thinning. First, we spatially correlated nodal centrality with the cortical thinning patterns in patients with MUD. Second, we examined the relationship between regional cortical thinning and the collective cortical thickness reduction of neighboring regions, weighted by the healthy brain connectome. The relationship between network-weighted cortical abnormality and clinical phenotype scores was estimated by partial least squares (PLS) regression. To assess the effects of drug use duration and dosage on network-based cortical thickness alterations, we performed clustering analysis on MUD subgroups using individual network-weighted cortical abnormality scores. Building on prior studies of neurodegenerative and psychiatric disorders, we hypothesized that cortical thickness reductions in MUD are shaped by connectome architecture. Specifically, brain regions with high network centrality may be more susceptible to morphometric alterations, and such abnormalities may propagate to connected areas via structural pathways.

## Materials and methods

### Study samples

The study recruited 139 individuals with MUD from the Kangda Voluntary Drug Rehabilitation Centre and Hunan Brain Hospital in Hunan Province, China. We also recruited 119 healthy controls (HCs) from local communities via online advertisements. All participants were right-handed and aged between 18 and 45 years. The patients with MUD met the Diagnostic and Statistical Manual of Mental Disorders, Fourth Edition, Text Revision (DSM-IV-TR) criteria for substance dependence (methamphetamine) as determined by the Structured Clinical Interview for DSM Disorders. Exclusion criteria included learning disabilities, neurological or psychiatric conditions (excluding substance-induced symptoms), history of traumatic brain injury (>10 minutes unconsciousness), familial predisposition to heritable mental disorders, recent neuromodulatory interventions within 3 months, pregnancy, MRI contraindications, and dependence on non-nicotine substances.

The entire study protocol was approved by the institutional review board of the Second Xiangya Hospital (No. S163, 2011) and Sir Run Run Shaw Hospital, Zhejiang University School of Medicine (No. 2023-826-01), in accordance with the Declaration of Helsinki (1975, revised 2008). Written informed consent was obtained from all participants prior to their involvement in the study.

### Clinical measurements

The sociodemographic characteristics and drug use patterns of each participant were evaluated, including sex, age, years of education, duration of drug use (in months), and lifetime drug consumption. Among the patients with MUD, we used a 10-point visual analogue scale to assess methamphetamine craving, where 0 represents the lowest craving level and 10 indicates the highest intensity of craving (Mezinskis, Honos-Webb, Kropp, & Somoza, 2001). Additionally, the severity of psychosis, depression, and anxiety symptoms was measured using the positive and negative syndrome scale (PANSS), the 24-item Hamilton depression rating scale (HDRS), and the 14-item Hamilton anxiety rating scale (HARS) (Kay, Fiszbein, & Opler, 1987; Maier, Buller, Philipp, & Heuser, 1988; Williams, 1988), respectively. To ensure the reliability of these assessments, all psychiatrists involved in the study underwent extensive training in administering PANSS, HDRS, and HARS before the study began.

### MRI data acquisition

The T1-weighted anatomical images were acquired using a Siemens Magnetom Trio 3 T scanner (Allegra; Siemens, Erlangen, Germany) with a 64-channel head coil. The scanning parameters were as follows: a 3D MPRAGE sequence with a 256 × 256 mm^2^ field of view, 1 mm slice thickness with no gap, repetition time (TR) of 2000 ms, repetition time (TE) of 3.7 ms, flip angle of 8, and a total of 176 slices.

### Image processing and cortical thickness calculation

All neuroimage data were preprocessed via FreeSurfer software (version 7.1, https://surfer.nmr.mgh.harvard.edu). The preprocessing of 3D-T1 images employed a standard auto-reconstruction algorithm that included several key steps: motion correction, intensity normalization, non-brain tissue removal, automated transformation into Talairach space, segmentation of white and gray matter, volumetric structure delineation, and tessellation. In addition, automated topology correction and surface deformation techniques were applied. Cortical thickness (CT) was estimated at each surface vertex by calculating the shortest distance between the gray and white matter surfaces. For each participant, CT measurements were obtained for 68 cortex regions according to the Desikan-Killiany parcellation atlas (Desikan et al., 2006). The quality of cortical parcellations was visually assessed, with histograms generated for all regions to enable a comprehensive visual examination. No subjects were excluded from the analysis.

We identified cortical thinning patterns associated with MUD by employing a general linear model to assess regional CT differences between patients and healthy controls. Covariates such as sex, age, years of education, and intracranial volume (ICV) were included in the analysis. Bonferroni correction was used for multiple comparisons across 68 brain regions, resulting in an adjusted significance threshold of p < 0.05/68. Statistical t-maps were extracted and converted to z statistics for following analysis.

### Structural, morphological, and functional network reconstruction

We utilized the ENIGMA toolbox (https://enigma-toolbox.readthedocs.io/en/latest/) to derive resting-state functional connectivity and structural connectivity matrices. These matrices, sourced from healthy adults (n = 207, 40% males, mean age = 28.73) in the Human Connectome Project (HCP; http://www.humanconnectome.org/), have been employed in prior studies exploring the impact of brain networks on cortical alterations (Georgiadis et al., 2024; Hettwer et al., 2022). A group-average functional connectivity matrix was generated by calculating pairwise correlations between 68 cortical regions of Desikan-Killiany atlas. Additionally, the group-average structural connectivity matrix was computed by applying a distance-dependent threshold to the number of streamlines connecting these regions. For more details, see Supplementary Materials.

For the morphological network analysis, CT measurements were obtained from 68 cortical regions, as defined by the Desikan-Killiany parcellation scheme, using data from our healthy control group. A group-level morphological network was constructed by calculating Pearson correlations between the CT values of each pair of regions. Fisher r-to-z was applied to the correlation coefficients to improve normality.

### Network analysis of cortical alterations

The modal stress model and nodal-neighbor common atrophy model were modeled the relationship between normal connectome across three modality and cortical thickness changes in MUD (Supplementary Figure S1). In the nodal stress model (Zhou et al., 2012), we predicted that higher degree centrality of a node would exhibit larger cortical thinning in MUD. Weighted degree centrality was calculated using structural, morphological, and functional connectivity data by summing the values of all cortico-cortical connections for each region, respectively. Spatial correlation method was used to examine relationship between MUD-related cortical thinning patterns (t-value map) and normative weighted degree centrality profiles using Pearson correlation coefficients. The significance of associations of nodal centrality and cortical thinning were tested by spatial autocorrelation permutation tests (spin tests) with 5000 times (Alexander-Bloch et al., 2018). Specifically, the centroids of the 68 cortical regions defined by the Desikan-Killiany atlas were projected onto a spherical surface. The surface was then randomly rotated across the sphere, preserving the spatial topology of the brain. After each rotation, regional values were reassigned according to the closest parcel on the rotated sphere. This process was repeated 5000 times to generate a null distribution that maintains spatial dependencies. The empirical p-value was computed as the proportion of permuted correlations that exceeded the observed correlation.

In the nodal-neighbor common atrophy model (Shafiei et al., 2020), we predicted that nodes exhibiting more severe cortical changes are connected to neighboring regions with greater cortical thinning. At the group level, we used healthy connectivity networks from three modalities to estimate the morphological abnormalities of the neighbors for each region. The structurally weighted neighbor cortical thinning level of a region was then calculated as the average cortical thinning values of all the brain regions structurally connected to that node:
(1)

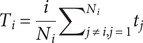

where *T_i_* represents the average neighbor cortical thinning value of structural neighbors of a given node *i*, *t_j_* is the cortical thinning of the *j*th neighbor structurally connected to node *i*, and *N_i_* is the total number of regions structurally connected to node *i.* Normalization by the term *N_i_* ensures that the estimated neighbor cortical thinning value is independent of the nodal degree. The calculation of neighbor cortical thinning excludes self-connections (i.e., *i* ≠ *j*).

The morphological-weighted connected neighbor cortical thinning level was assessed using Equation (1), with the exception that cortical thinning values were weighted by the strength of morphological connectivity between nodes *i* and *j* (*SMN_ij_*):
(2)

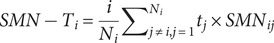

where *SMN-T_i_* represents the average neighbor cortical thinning value of morphologically defined neighbors of a given node *i*, and *SMN_ij_* is the mean morphological connectivity between nodes *i* and *j.*

Similarly, the functional-weighted connected neighbor cortical thinning level was assessed using Equation (2), with the exception that cortical thinning values were weighted by the strength of functional connectivity between nodes i and j (*FC_ij_*):
(3)

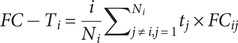

where *FC-T_i_* represents the average neighbor cortical thinning value of morphologically defined neighbors of a given node *i*, and *FC_ij_* is the mean functional connectivity between nodes *i* and *j.*

For structural-, morphological- and functional-weighted neighbor cortical thinning estimations, neighbors of a region were determined as a node structurally connected to that region. Pearson correlation coefficients were employed to assess the relationship between nodal cortical thinning and its structurally, morphological- and functionally weighted connected neighbors cortical changes level, respectively. The spatial autocorrelation-preserving permutation tests was determined the significance of associations of nodal centrality and cortical thinning (5000 repetitions).

### Epicenter identification

To identify potential epicenters of cortical thinning in MUD, we adopted a nodal-neighbor cortical thinning ranking method (Shafiei et al., 2020). Specifically, for each brain region, we first ranked the degree of cortical thinning (based on the group-level MUD versus control thickness difference) in ascending order. Separately, for each region, we computed the morphology-weighted average cortical thinning level of its structurally connected neighbors using the morphological covariance network and ranked these values in the same way. The final epicenter likelihood for each region was determined by averaging its own atrophy rank and its neighbor-based rank. Regions with the lowest mean ranks were considered the most probable epicenters, as they showed high levels of cortical thinning themselves and were surrounded by similarly affected neighbors. To assess the statistical significance of these rankings, we employed a spin permutation test with 5000 repetitions, which preserves the spatial autocorrelation structure of the cortical surface by rotating the cortical thinning map. Given that the morphological network best explained cortical thinning patterns in both the nodal stress and nodal-neighbor models (see Results), the epicenter analysis was conducted based on morphology-weighted neighbor thinning values.

### Individual network-weighted cortical abnormality model

To examine the associations between network-weighted cortical alterations (participants × brain regions) and clinical factors (participants × measures) in patients, a multivariate statistical algorithm, PLS regression, was employed (McIntosh & Mišić, 2013). Personalized network-weighted cortical abnormality was defined using connectome-weighted cortical thinning. Specifically, we estimated individual cortical thinning maps for patients with MUD by measuring deviations from the normal distribution. A multiple regression model was developed to predict regional cortical thickness, incorporating sex, age, education level, and ICV as covariates. This model was then applied to estimate the cortical thickness for each patient with MUD. Regional W-scores were generated by calculating the difference between the observed and predicted cortical thickness, divided by the standard error of the model fit in controls, providing individualized cortical thickness maps. A positive W-score means cortical thinning in patients with MUD in relative to HC and vice versa. Personalized network-weighted cortical abnormality of a region was determined by the normalized *W* score of all regions that were connected to region *i* by a morphological connection.
(4)

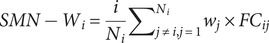

where *SMN-W_i_* represents the individual neighbor cortical thinning value of morphologically defined neighbors of a given node *i*, and *W* is individualized cortical thickness thinning.

PLS analysis was performed to investigate the association between individual network-weighted cortical abnormalities and clinical factors (including craving, PANSS, HARS, and HDRS). Network-weighted cortical abnormality maps served as predictor variables, while clinical scale scores were used as response variables. The significance of the variance explained by the PLS components was evaluated using permutation test with 5000 iterations. To model the effect of brain atrophy on clinical symptoms, Pearson correlation analyses were performed between network-weighted cortical abnormalities (brain scores) and the behavioral phenotypes (behavior scores) identified in the significant latent variable. The behavior loadings are denoted by the Pearson correlation between behavior phenotypic data and the brain scores.

### Associations of network-based cortical abnormality and drug use

Previous studies have indicated that network-level gray matter abnormalities progress from the early stages of illness to more severe stages in neurodegenerative and psychiatric diseases (Chopra et al., 2023; Wannan et al., 2019; Zhou et al., 2012). Building on these findings, we hypothesized that drug use, such as duration and dose of drug, might influence cortical thickness alterations at the network level, resulting in heterogeneous subgroups. To investigate the impact of drug use on network-based cortical thickness changes, we performed k-means clustering to identify subgroups related to drug use. K-means clustering divides the dataset into k non-overlapping groups by minimizing variance within clusters and maximizing differences between clusters, based on a predefined distance metric. This method has been widely used in psychiatric research for data-driven biotype discovery (Ha et al., 2023; Li et al., 2024). We first constructed a 139 × 139 similarity matrix by calculating the Pearson correlation coefficients between SMN-weighted cortical thinning profiles for all MUD participants. This matrix captures the spatial similarity in cortical thinning patterns between every pair of individuals, where higher values indicate greater similarity. Based on this similarity matrix, k-means clustering using Euclidean distance were applied to identify potential subgroups. To enhance robustness and reproducibility, the clustering procedure was repeated 100 times with random initializations. The optimal number of clusters (k) was determined by calculating silhouette scores for values of k ranging from 2 to 10. The differences in methamphetamine duration and dose between the subgroups were assessed using a two-sample test.

## Results

### Sample characteristics

Table 1 presents the sociodemographic characteristics of patients with MUD and HC. There was no significant group difference in sex and age between individuals with MUD and HC. However, patients with MUD showed lower education level than HC. Patients with MUD showed higher anxiety and depression level in relative to HC.

**Table 1.** The demographic and clinical characteristics of patients with methamphetamine use disorder and HC

	MUD	HC	x/t	p
Number	139	119		
Sex (Male/Female)	122/17	99/20	1.09	0.30
Age (Mean ± SD, years)	29.12 ± 5.40	28.31 ± 5.41	1.20	0.23
Education (Mean ± SD, years)	11.76 ± 2.66	14.55 ± 3.31	−7.51	**<0.05**
Duration (months)	52.22 ± 30.63			
Total dose estimated values	74.36 ± 123.98			
Craving (VAS)	2.47 ± 2.61			
PANSS				
P	18.29 ± 7.62			
N	19.85 ± 7.42			
G	39.47 ± 10.58			
T	77.07 ± 22.94			
HARS	16.28 ± 8.15	3.71 ± 4.69	12.960	**<0.05**
HDRS	26.58 ± 14.73	4.07 ± 7.17	13.159	**<0.05**

*Note*: Data are showed as the mean ± SD. HC, healthy control; HARS, Hamilton anxiety rating scale; HDRS, Hamilton depression rating scale; MUD, methamphetamine use disorder; PANSS, positive and negative syndrome scale; VAS, visual analogue scale.

### Cortical thickness reductions in patients with MUD

**Figure 1.**
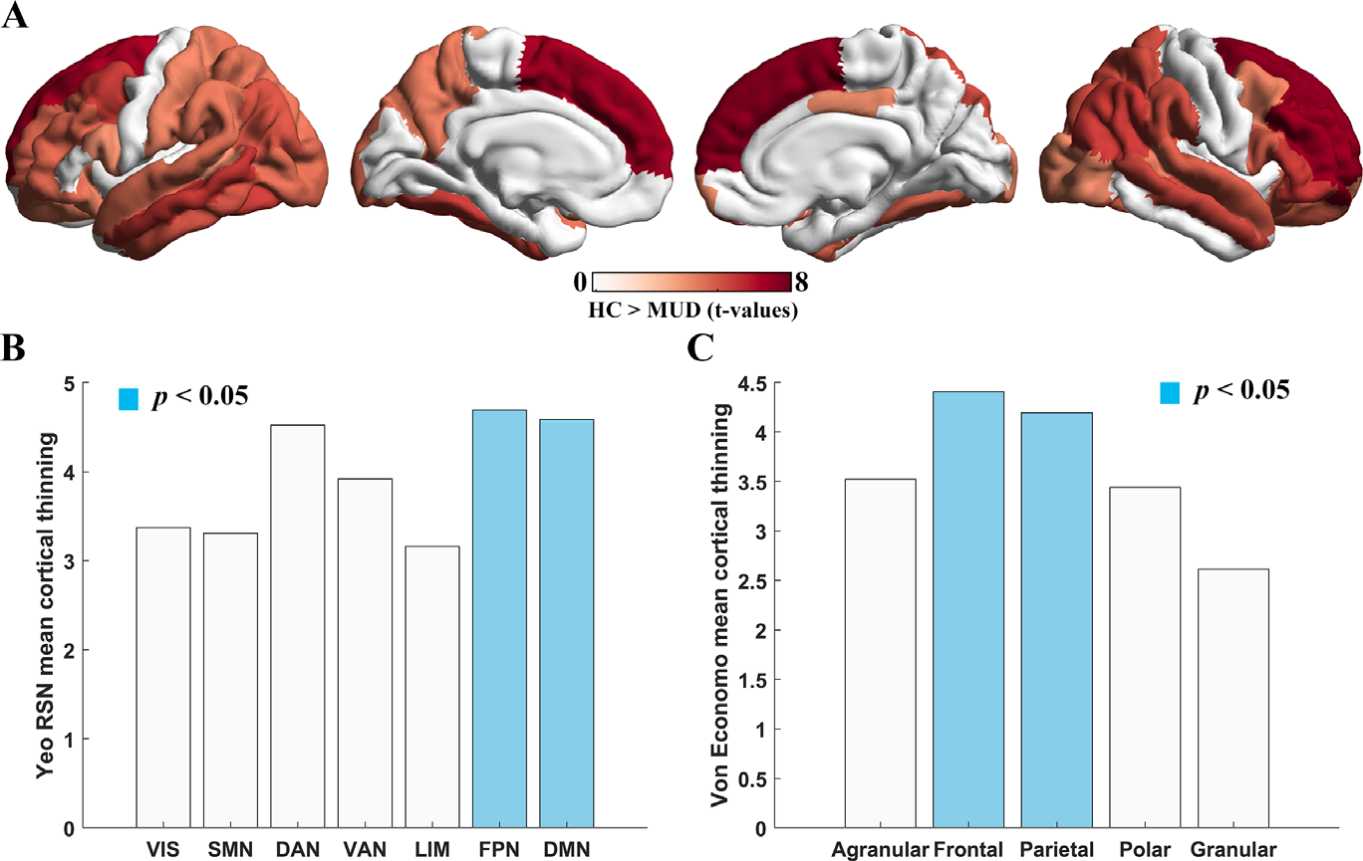
Cortical thickness reductions in patients with methamphetamine use disorder (MUD). (A) Maps displaying cortical morphological changes in both MUD patients and the control group. (B) Mean cortical alteration values assessed within resting-state networks based on the Yeo atlas. (C) Mean cortical alteration values calculated using the Von Economo-Koskinas atlas. The brain networks according to the Yeo atlas include: VIS (visual network), SMN (somatomotor network), DAN (dorsal attention network), FPN (frontoparietal network), VAN (ventral attention network), and DMN (default mode network).

Compared to HC, a total of 28 cortex regions were found to exhibit significant cortical thickness thinning in patients with MUD (p < 0.05, Bonferroni corrected). We did not find any increased cortical thickness in methamphetamine dependent groups. As illustrated in Figure 1, pronounced cortical thinning was observed in the frontal and temporal lobes, particularly in the superior frontal gyrus and middle temporal gyrus. To investigate whether this cortical thinning was predominantly associated with specific brain networks, we employed two brain atlases: (1) the Yeo atlas (Yeo et al., 2011), defined by intrinsic functional connectivity, and (2) the Von Economo-Koskinas atlas (Scholtens, de Reus, de Lange, Schmidt, & van den Heuvel, 2018), based on cytoarchitectonic classes. Mean cortical alteration values were calculated within each brain system according to both atlases. A permutation test with 5000 repetitions was used to assess the significance of cortical alterations at the network level. The results revealed that patients with MUD exhibited greater cortical thinning in the frontoparietal (FPN) and DMNs. For the Von Economo-Koskinas atlas, more significant cortical changes were observed within the frontal and parietal cytoarchitectonic classes.

### Brain connectome shape cortical thinning

**Figure 2.**
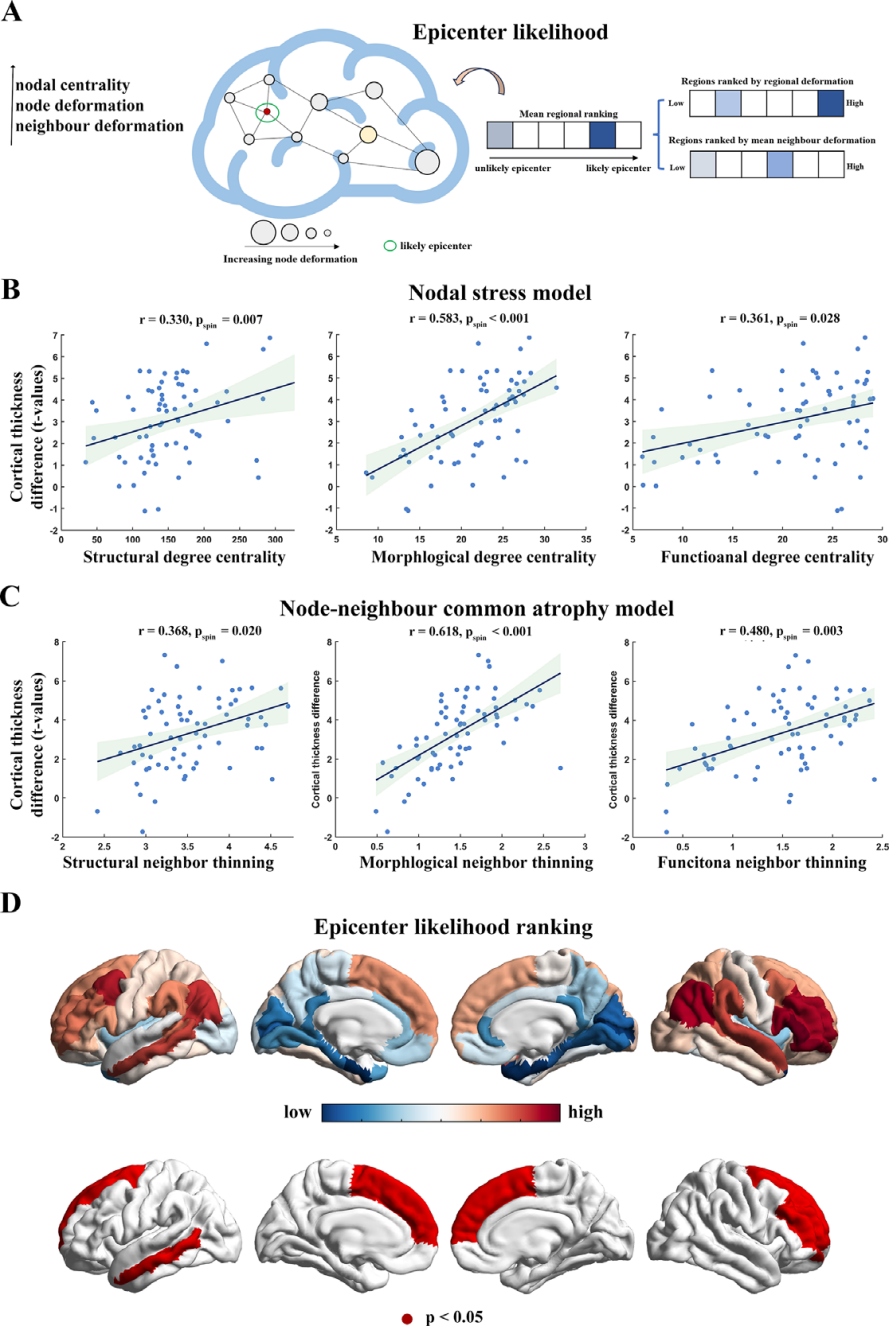
Network-based cortical thickness alterations. If regional morphometric changes depend on network connectivity, nodes with higher morphometric alterations exhibit high levels of normative network centrality and are connected to neighbors with greater nodal atrophy. (A) Schematic of epicenter identification: A region was defined as an epicenter if it exhibited high cortical thickness changes, and its connected neighbors also showed significant changes. (B) The T-value map of cortical thickness alterations is associated with structural, morphological, and functional connectivity of degree centrality. (C) Nodal thinning is related to alterations in its neighbors, defined by structural connectivity, and weighted by both morphological and functional connectivity. (D) The spatial distribution of the putative epicenters in MUD is shown. The upper panel illustrates the mean rankings mapped onto the brain surface, while the lower panel depicts the statistical significance of these rankings based on spin tests.

We employed two models to investigate the relationship between brain networks and cortical morphological alterations in patients with MUD. As described in the Figure 2, in the stress nodal model, we found that greater nodal degree centrality was significantly associated with more pronounced cortical thickness changes. This significant relationship was observed across different brain network modalities, including structural (r = 0.330, p_spin_ = 0.007), morphological (r = 0.583, p_spin_ < 0.001), and functional (r = 0.361, p_spin_ = 0.028) degree centrality. In the nodal-neighbor common atrophy model, cortical thinning in a specific region was significantly linked to the cortical thinning of its connected neighbors across structural (r = 0.368, p_spin_ = 0.020), morphological (r = 0.618, p_spin_ < 0.001), and functional (r = 0.480, p_spin_ = 0.003).

### Disease of epicenters

Using a network-based node-neighbor ranking method, we identified an epicenter likelihood pattern (Figure 2A). This method determines the epicenter probability of a region by calculating the mean ranking based on both the nodal cortical thinning value and the thinning values of its neighboring regions. We found that regions in the bilateral superior frontal gyrus, the right rostral middle frontal gyrus, the right pars orbitalis, and the left middle temporal gyrus exhibited significantly higher mean rankings (Figure 2D, p_spin_ < 0.001). This finding was consistent across both structural- and functional-weighted neighbor thinning analyses.

### Mapping individual network-weighted cortical thinning on clinical symptoms

**Figure 3.**
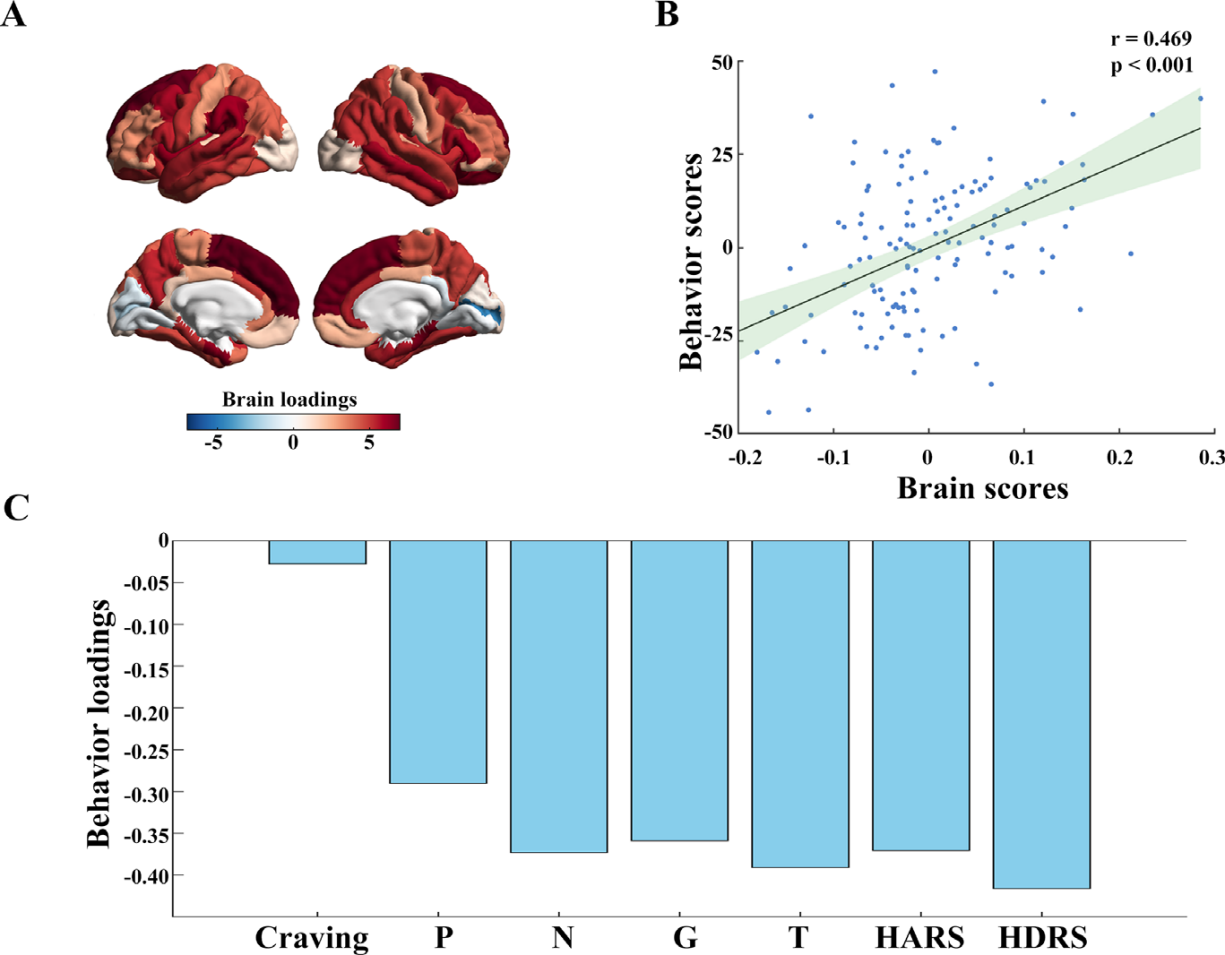
The associations of individual network-weighted cortical alterations (W-scores) and clinical symptoms. (A) The first latent variable derived from a partial least squares (PLS) regression analysis linking individual W-scores. (B) The relationship between brain scores and behavior scores, where brain scores are defined by expression of individual W-scores on the first latent variable, and behavior scores represent the behavioral phenotype on the first latent variable. (C) The bar plot depicts the behavioral loadings, which were calculated based on the relationship between behavioral phenotypic data and brain scores.

The PLS analysis identified the first latent variable, which accounted for 11.66% of the covariance between network-based cortical abnormality maps and clinical scores (p = 0.043, permutation test). As shown in Figure 3, this latent variable highlighted a cortical thinning pattern predominantly in prefrontal areas, closely resembling the epicenter likelihood distribution. Individuals exhibiting this thinning pattern experienced more severe psychosis, anxiety, and depression symptoms. This pattern was not related to craving.

### Network-based cortical thinning identified two MUD biotypes

**Figure 4.**
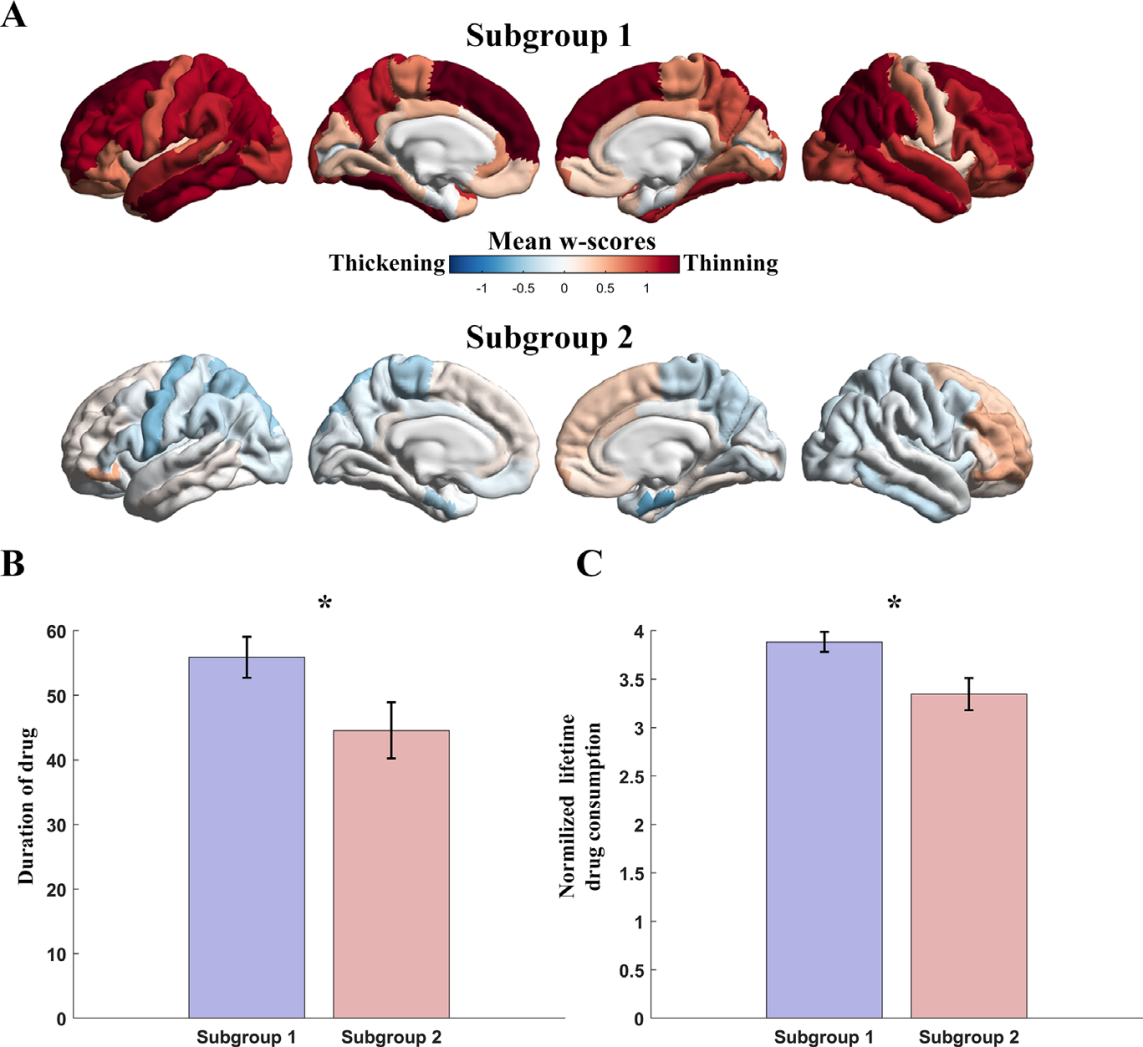
Clustering analysis. (A) The distribution of w-scores in subgroup 1 and subgroup. (B) The group difference for each subgroup in the duration of methamphetamine use. (C) The group difference between two subgroups in the total dose of methamphetamine use. Error bars represent the standard deviation of the data.

We employed an unsupervised approach to investigate the impact of drug use on cortical thinning at the network level. As shown in Figure 4, K-means clustering analysis identified two distinct MUD biotypes. Subgroup 1 (n = 94) displayed patterns of cortical thinning, whereas subgroup 2 (n = 45) exhibited a tendency toward cortical thickening. Subgroup 1 had significantly longer durations of methamphetamine use (t = 2.064, p = 0.040) and higher doses of methamphetamine use (t = 2.896, p = 0.004) compared to subgroup 2.

## Discussion

The current study employed both the nodal stress model and the nodal-neighbor common atrophy model to investigate cortical thinning patterns in patients with MUD at the network level. The findings revealed that cortical thinning was constrained by healthy brain networks across distinct modalities. We observed widespread cortical thickness reduction in patients with MUD, accumulating in specific systems. Moreover, nodes exhibiting greater cortical thinning were associated with higher nodal degree centrality and connected neighbors’ cortical alterations. The frontal and temporal regions emerged as key epicenters. The individual network-based patterns of cortical alteration correlated with clinical symptom variability, distinguishing MUD subtypes associated with drug use.

Our findings align with previous studies (Joo et al., 2023; Lyoo et al., 2015), showing widespread cortical thickness reduction in MUD patients, especially in the prefrontal and temporal areas. Cortical thinning was organized by specific functional brain networks, including the FPN and DMN. It was also structured by cytoarchitectonically defined frontal and parietal regions. These loci anchored within these functional networks, mainly in the prefrontal areas, were linked to inhibitory control and motivation (Friedman & Robbins, 2022; Munakata et al., 2011). Dysfunctions in these behaviors contribute to the initiation and maintenance of addiction (Ceceli, Bradberry, & Goldstein, 2022; Goldstein & Volkow, 2011). Combining these insights with epicenter analysis, we identified the dorsolateral prefrontal cortex as the likely spatial origin of cortical thinning in MUD, serving as a central hub for the network-driven spread of cortical changes. This suggested that damage to the prefrontal cortex is a critical factor in both vulnerability to and resilience against drug addiction (Ersche et al., 2020).

Our results further elucidated the relationship between brain networks and cortical thickness alterations, providing evidence that cortical morphological changes in MUD may be constrained by network architecture through both the nodal stress and nodal-neighbor models. According to the nodal stress hypothesis, highly interconnected nodes were positively correlated with greater degrees of deformation, suggesting that atrophy in patients with MUD is shaped by the topology of the brain connectome. In line with our findings, previous studies have reported alterations in small-world properties and modularity across various substance use disorders, including nicotine (Fan et al., 2023), alcohol (Ottino-González & Garavan, 2022), methamphetamine (Liu et al., 2022), and heroin (Yuan et al., 2010). Additionally, cortical thickness alterations in MUD were influenced by white matter architecture, as significant relationships were found between nodal cortical changes and those of their connected neighbors. This may be due to impaired transneuronal transport of trophic factors, leading to brain tissue loss (Zalesky et al., 2015). Brain-behavior analysis revealed that individuals with greater deviations in cortical thickness in the prefrontal areas from the normal model were associated with more severe clinical symptoms, such as psychosis and negative phenotypes. This suggests that network-based morphological alterations not only help trace disease epicenters but also map cortical alterations underlying clinical symptoms (Li et al., 2023). Altogether, these findings indirectly support the idea that cortical morphological alterations in MUD patients align with the transneuronal network spread model, providing an explanation for the widespread, irregular, and discontinuous pattern of cortical thickness reduction in MUD. It is important to note that our findings remain correlational, making it difficult to disentangle the causal relationship between cortical thickness and network architecture in MUD. Beyond network-level factors, accumulating evidence suggests that neurobiological processes such as neuroinflammation and excitotoxicity may also play a role (Li et al., 2024). For example, an early PET study using a Translocator Protein (TSPO) tracer reported elevated microglial activation (an indicator of neuroinflammation) in the midbrain and insular cortex of individuals with MUD (Sekine et al., 2008). Decreased glutamine levels have been observed in the medial prefrontal cortex of MUD individuals relative to HC, which may reflect altered glutamatergic neurotransmission (Wu et al., 2018). These findings underscore the importance of future multimodal studies to elucidate how inflammatory and excitotoxic processes interact with structural networks in driving cortical degeneration in MUD.

We further identified two distinct biotypes of MUD based on individual cortical thickness thinning scores at the network level. Consistent with our hypothesis, these two biotypes exhibited significant differences in drug use variables, such as the duration and dose of methamphetamine use. Individuals with longer histories and higher doses of methamphetamine use showed more extensive cortical alterations, suggesting a progressive network spread of gray matter changes in MUD. The dose-effect analysis further supports this notion, as it revealed that global mean cortical thickness in chronic methamphetamine users was significantly negatively correlated with the lifetime dose of drug use (Joo et al., 2023; Lyoo et al., 2015). These findings are reminiscent of observations in neurodegenerative disorders and schizophrenia (Chopra et al., 2023; Leuzy et al., 2023; Wannan et al., 2019), where patients in the later stages of illness tend to experience greater brain structural alterations. This suggests that the severity and duration of drug exposure play a critical role in the extent of cortical thinning observed in MUD.

The current study has several limitations. First, our imaging findings were derived from cross-sectional data, which limit our ability to make causal inferences about the progression and potential propagation of cortical thinning in MUD. As such, we cannot determine whether the observed morphometric alterations reflect consequences of prolonged drug exposure, predisposing factors, or dynamic disease processes. To validate the network spread hypothesis and clarify the temporal sequence of structural changes in relation to brain connectome architecture, future longitudinal studies are essential. Second, we derived group-level normative structural and functional connectivity matrices from HCP samples. Future research should investigate individual connectivity-based models of atrophy to evaluate how disease-specific connectomes influence brain atrophy. Third, all findings are based on a single type of addictive drug and a single dataset. Although we used a relatively large sample, the results should ideally be validated independently using a distinct MUD dataset. Additionally, the generalizability of network spreading of gray matter alterations in addiction should be further examined using different types of drugs. Fourth, individuals with MUD exhibited lower education levels than HC, which could potentially influence cortical thickness measurements. Previous studies have shown that variations in socioeconomic status are a key risk factor for substance use disorders and are associated with alterations in brain structure and function (Evans-Lacko et al., 2018; Tian et al., 2021). Future research is encouraged to further investigate the specific impact of educational attainment and other sociodemographic variables on brain alterations in MUD.

In conclusion, our results suggest that cortical connectivity networks can explain the irregular distribution of cortical thickness reductions in MUD. We identified the prefrontal areas as the likeliest ‘disease epicenters’. Network-based cortical thickness reductions were also correlated with the severity of individual clinical symptoms and could distinguish distinct diagnostic MUD subtypes associated with drug use. These findings provide network mechanistic insights into cortical morphological changes in MUD and addiction.

## Supporting information

Sun et al. supplementary material Sun et al. supplementary material

## Supplementary material

The supplementary material for this article can be found at http://doi.org/10.1017/S0033291725102067.
