# Supplementary material for: Network-dependent cortical thickness reductions following chronic methamphetamine use
Source: Psychol Med. 2025 Oct 3;55:e296. doi: 10.1017/S0033291725102067 (PMC12527499; doi:10.1017/S0033291725102067)
Supplement: Sun et al. supplementary material [file S0033291725102067sup001.docx]

**Supplementary Material**

**Network Dependent Cortical Thickness Reductions Following Chronic Methamphetamine use**

Yunkai Sun^1^, Jun Wang^1^, Jinsong Tang^1^, Yanhui Liao^1*^

1. Department of Psychiatry, Sir Run Run Shaw Hospital, Zhejiang University School of Medicine, Hangzhou, Zhejiang, P. R. China

^*^Corresponding Author: Yanhui Liao, 3 East Qingchun Road, Hangzhou, Zhejiang, China (310016). E-mail: liaoyanhui@zju.edu.cn, Tel or Fax: 0571-87887070.

**Supplementary Methods**

**HCP MRI data acquisition and preprocessing**

HCP data were acquired on a Siemens Skyra 3T. The T1-weighted anatomical images were acquired using MPRAGE sequence with the following scanning parameters: repetition time (TR) = 2400 ms, echo time (TE) = 2.14 ms, field of view (FOV) = 224 × 224 mm2, voxel size = 0.7 mm3, 256 slices. The functional images were obtained by EPI sequence with the following scanning parameters: TR = 720 ms, TE = 33.1 ms, FOV = 208 × 180 mm^2^, voxel size = 2 mm3, 72 slices. Diffusion tensor images were acquired using spin-echo EPI sequence with TR = 5520 ms, TE = 89.5 ms, FOV = 210 × 180mm^2^, voxel size = 1.25 mm^3^, b-value = 1000/2000/3000 s/mm^2^, 270 diffusion directions, 18 b_0_ images).

Resting-state fMRI data were subjected to multiple preprocessing steps, including corrections for head motion and distortion, magnetic field bias correction, skull stripping, and intensity normalization. The data were subsequently mapped to MNI152 space. To eliminate noise components, ICA-FIX was applied, removing signals attributed to head motion, white matter, cardiac pulsation, arteries, and large veins. The preprocessed time series were then transformed into standard gray ordinate space using a cortical ribbon-constrained volume-to-surface mapping algorithm, after which they were concatenated into a unified time series.

For diffusion MRI data, preprocessing involved b_0_ intensity normalization along with corrections for susceptibility-induced distortions, eddy currents, and head motion. Functional and structural data with high resolution were finally parcellated based on the Desikan-Killiany atlas.

**Functional and structural connectivity matrix construction**

Functional connectivity matrices were created by calculating pairwise correlations between the time series of 68 cortical regions, with negative values set to zero. These subject-specific connectivity matrices were z-transformed and averaged across participants to construct a group-level functional connectome.

For structural connectivity matrices, constrained tractography was performed using tissue types derived from T1-weighted images, including cortical and subcortical gray matter, white matter, and cerebrospinal fluid. Multi-shell and multi-tissue response functions were estimated, followed by constrained spherical deconvolution and intensity normalization. An initial tractogram consisting of 40 million streamlines was generated, with a maximum tract length of 250 mm and a fractional anisotropy threshold of 0.06. Spherical-deconvolution informed filtering of tractograms (SIFT2) was then applied to weight the streamlines based on cross-sectional multipliers. To construct normative subject-specific connectivity matrices, the reconstructed streamlines were mapped to the 68 cortical regions defined by the Desikan-Killiany atlas. These structural connectivity matrices were derived from preprocessed diffusion MRI data using MRtrix3. The group-level normative structural connectome was generated by applying distance-dependent thresholding to maintain the edge length distribution across individual participants. Log transformation was applied to reduce variance in connectivity strength. Structural connectivity was thus quantified as the number of streamlines connecting two regions, representing fiber density.


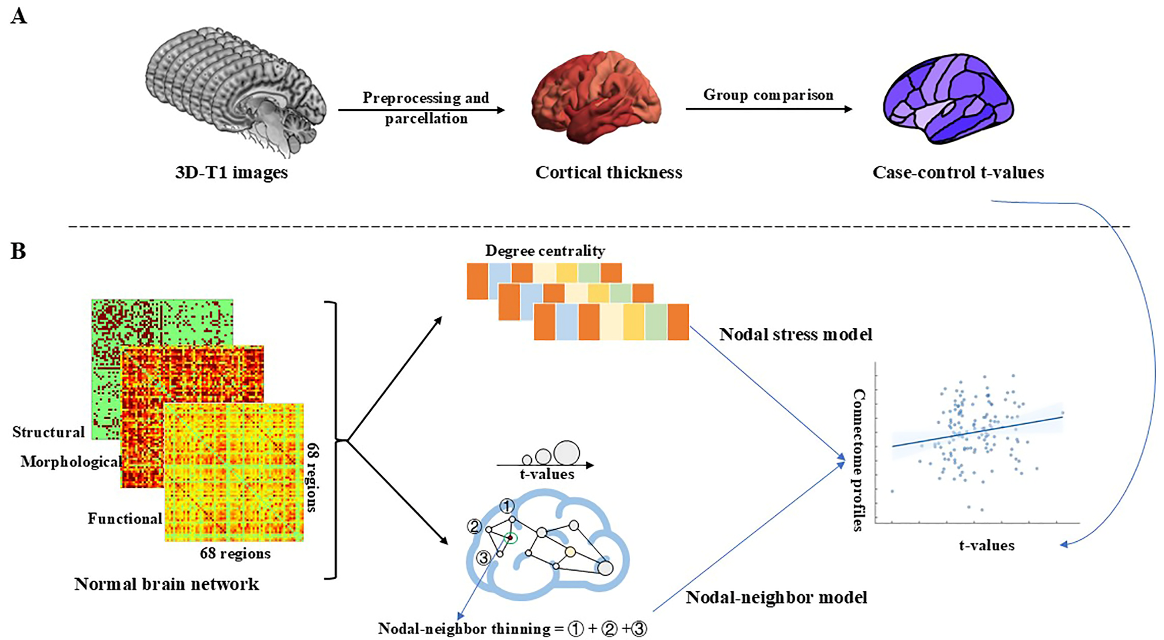


**Figure S1. Flowchart of the network-based cortical thickness alteration analysis. (A) Cortical thickness was extracted for each subject using FreeSurfer with default parameters. The Desikan-Killiany (DK) atlas was applied to obtain thickness measures for 68 cortical regions. A general linear model (GLM) was then used to assess group differences in cortical thickness between individuals with methamphetamine use disorder (MUD) and healthy controls, controlling for age, sex, years of education, and intracranial volume (ICV). (B) Two network-based models—the nodal stress model and the nodal-neighbor model—were used to examine the relationship between cortical thinning in MUD and the healthy brain connectome. Structural, morphological, and functional connectivity matrices were derived from healthy participants. For the nodal stress model, multimodal weighted degree centrality was computed and correlated with MUD-related cortical thickness alterations (t-values). For the nodal-neighbor model, the average cortical thinning of structurally, morphologically, and functionally connected neighboring regions was calculated and then correlated with MUD-related cortical thickness changes. The statistical significance of both models was assessed using spatial autocorrelation-preserving permutation tests.**
